# The importance of diastolic dysfunction in the development of weaning-induced pulmonary oedema

**DOI:** 10.1186/s13054-017-1613-5

**Published:** 2017-02-11

**Authors:** Filippo Sanfilippo, Cristina Santonocito, Gaetano Burgio, Antonio Arcadipane

**Affiliations:** Department of Anesthesia and Intensive Care, IRCCS-ISMETT (Istituto Mediterraneo per i Trapianti e Terapie ad alta specializzazione), Via Tricomi 5, 90127 Palermo, Italy

The group of Prof. Monnet et al. [1] elegantly described the characteristics of patients failing spontaneous breathing trials (SBTs; n = 128/283, 45%), confirming that a large proportion of weaning failures (59%) are associated with weaning-induced pulmonary oedema (WiPO). Three factors were independently associated with WiPO during SBT: chronic obstructive pulmonary disease, obesity and “structural cardiopathy”.

However, we believe this study also deserves comment for the contribution of LV diastolic dysfunction (LVDD) in cases of WiPO. Despite patients with WiPO having similar LV ejection fraction to those without (61 versus 57%, *p* = 0.76), they had a higher E/E’ ratio (10.5 versus 8.8, *p* < 0.01), a parameter strongly associated with LVDD [2]. Furthermore, among patients with cardiac output (CO) monitoring in place during the SBT (n = 85/283), those developing WiPO showed a significant increase in global end-diastolic volume (~200 ml, +22% from baseline), while this parameter remained unchanged when WiPO did not occur. Interestingly, the vast majority of patients experiencing WiPO (n = 28/30) had preload-independence after a passive leg rising (PLR) test and, on the contrary, the PLR test showed preload-dependence in all the patients that did not experience WiPO (n = 55/55). The authors also reported that when preload-independence persisted despite fluid removal, most of the patients again showed WiPO on the following SBT, while a change to a preload-dependence condition was associated with a high rate of successful weaning.

Taken together, such findings emphasize the importance of LVDD as a contributor to WiPO. The higher venous return during the shift from positive to negative pressure ventilation determines unfavourable LV loading conditions, which may be poorly tolerated in the context of LVDD.

The importance of LVDD is not surprising since it has been associated with weaning failure [3, 4] and also with mortality in sepsis [5]. Of note, the authors report a higher incidence of septic-related cardiomyopathy in patients with WiPO (17 versus 2%, *p* = 0.01) [1].

We ask the authors to share their opinion on this aspect and to provide the E’ values comparing patients with or without WiPO, since the recently published guidelines have emphasized also the role of E’ when assessing LVDD [2].

On a separate note, another interesting finding that may deserve further comment is that patients with CO monitoring had a trend towards lower SBT failure (n = 45/85) compared to those with no CO monitoring (n = 83/198; *p* = 0.09, not reported). Was the CO monitoring intentionally used to keep the patient in a “safely dry” condition?

## Abbreviations

CO: Cardiac output
LV: Left ventricular
LVDD: Left ventricular diastolic dysfunction
SBT: Spontaneous breathing trial
WiPO: Weaning-induced pulmonary oedema
